# Molecular connectomics: Placing cells into morphological tissue context

**DOI:** 10.1371/journal.pbio.3002803

**Published:** 2024-08-26

**Authors:** Stathis Megas, Nadav Yayon, Kerstin B. Meyer, Sarah A. Teichmann

**Affiliations:** 1 Cambridge Stem Cell Institute, Jeffrey Cheah Biomedical Centre, Cambridge Biomedical Campus, University of Cambridge, Cambridge, United Kingdom; 2 Wellcome Sanger Institute, Wellcome Genome Campus, Hinxton, Cambridge, United Kingdom; 3 Department of Medicine, University of Cambridge, Cambridge, United Kingdom; 4 European Bioinformatics Institute (EMBL-EBI), Wellcome Genome Campus, Hinxton, Cambridge, United Kingdom; 5 Co-director of CIFAR Macmillan Multi-scale Human Program, Toronto, Canada

## Abstract

This Perspective introduces "molecular connectomics" to link molecular and morphological cell features in three dimensions across scales, using machine learning and artificial intelligence to reveal emergent properties of complex biological systems.

## Teaser

Here we propose "molecular connectomics" to link molecular and morphological cell features in three dimensions across scales, using machine learning and artificial intelligence to reveal emergent properties of complex biological systems.

Recent progress in the field of single-cell biology has led to an explosion of data providing molecular descriptions of individual cells and cell types, which are increasingly mapped into their tissue context. Dissociated single cells are now routinely profiled by combining multiple molecular features—mainly, the transcriptome, metabolome, and proteome. However, what is as yet missing is a better link to the morphological description of cells and tissues. Connectomics is a longstanding concept in neuroscience that has been applied to understand how individual neurons are connected to form functional networks.

**Fig 1:**
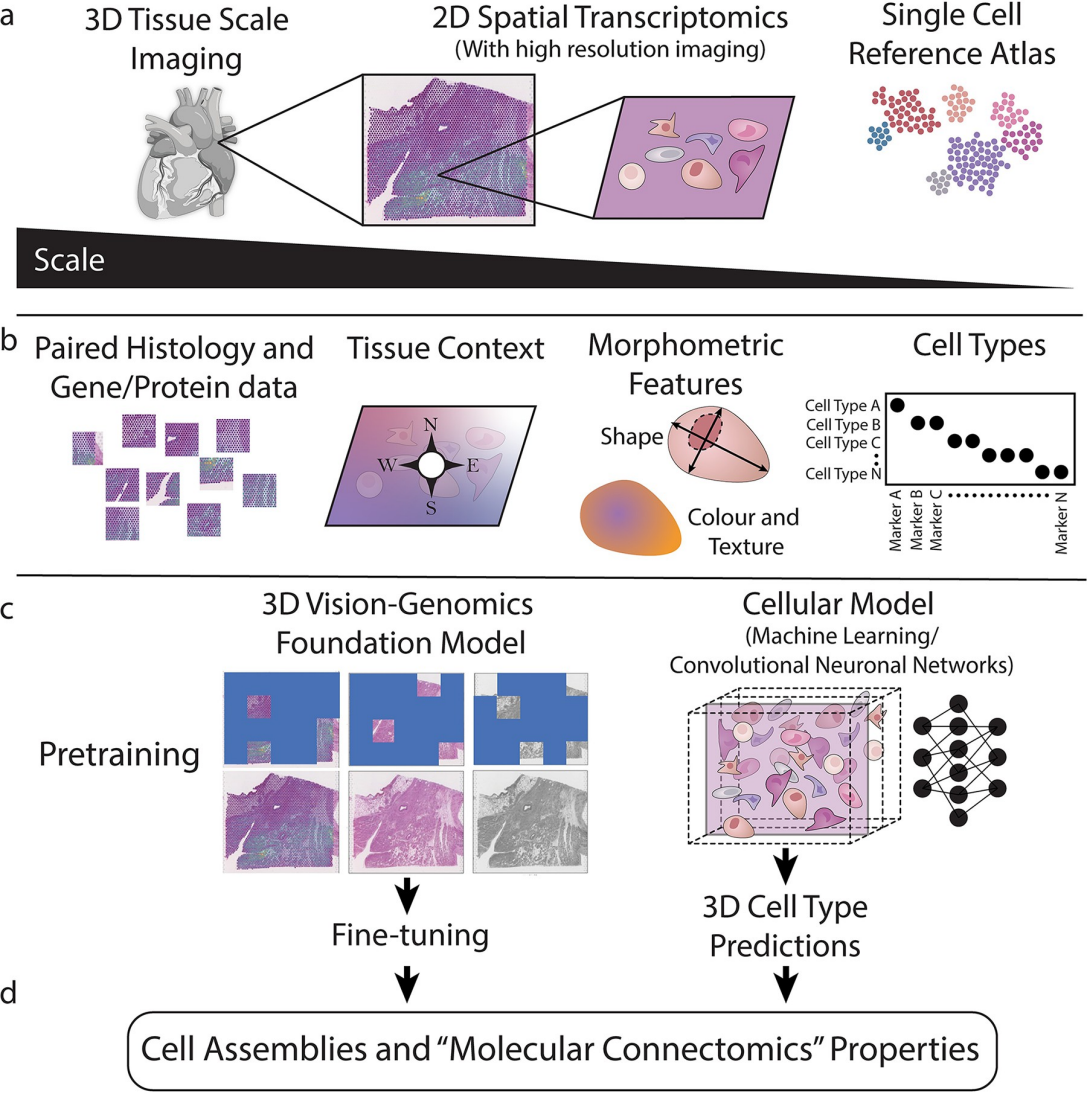
Molecular connectomics aims to provide in-depth profiling of molecular and morphological features of cell assemblies within their 3D micro- and macro-environments. **a)** The basic datasets are composed of spatial and single cell omics together with 3D large scale, high resolution imaging. **b)** Augmented omics data along with cell morphometric within the tissue contexts enable the training of models that can predict cell types in 2D space. **c)** Linking all modalities with tissue scale 3 dimensional data (e.g. HIP-CT) can be approached by task specific or foundation models trained via multi-modal masking and virtual 2D sectioning. **d)** The success of these models will lead to the discovery and emergence of “molecular connectomics” properties in true 3D space.

Here we propose the term “molecular connectomics” for all research in tissues that aims to first provide in-depth profiling of molecular features of the cell as well as its three-dimensional (3D) micro- and macro-environment, and second, to link molecular and morphological features using novel computational methods (Fig 1). We propose that, as molecular interactions of cells are studied at increasing scales, new emergent properties will give insights into whole-body function. For this, molecular analyses will need to harness recent developments in machine learning and artificial intelligence and account for the 3D nature of tissues across scales. Unlike classical connectomics in neuroscience, molecular connectomics does not necessarily require nanometer resolution (e.g., electron microscopy, which is critical in determining synaptic connections) to infer cell-to-cell communication between cells with more stereotypical and regular geometries.

However, obtaining cellular resolution, isotropic 3D measurements of entire biological systems at full molecular breadth is currently impossible. So, how can we generate molecular connectomics data, and what are the major computational challenges and methods?

Commercial spatial transcriptomics methods such as VisiumHD or StereoSeq are now able to measure the whole transcriptome at single-cell or even subcellular resolution [1], although whole transcriptome resolution can only achieve lower sensitivity per gene. Alternatively, protocols such as MERFISH, Molecular Cartography, or Xenium profile selected gene panels. As the size of gene panels is increasing, the two different approaches now both generate high-quality, 2D-resolved single-cell data. Further layers of data can be added by charting chromatin accessibility in space (e.g., slide-tag [2]) or by multi-modal tissue profiling [3]. This is complemented by classic immunohistochemistry, which is being used at increasing multiplexity (e.g., by CODEX or IBEX). All of these methods generate 2D molecular data that can be linked to histological hematoxylin and eosin staining data. Immunohistochemistry imaging can also generate 3D data, via tissue clearing and sophisticated microscopy, but usually for far fewer features at a time. A few specialized technologies now yield true 3D data, such as cell 3D positioning by optical encoding (C3PO) for genomics for limited numbers of genes or hierarchical phase-contrast computed tomography (HiP-CT) [4], which is able to generate high-resolution 3D imaging over large scales, but as yet without any link to molecular data. Therefore, to decipher molecular connectomics, a key challenge remains the translation of 2D molecular data into 3D morphological space.

Currently the most straightforward method of profiling histology and transcriptomics in 3D is by stacking consecutive layers of 2D technologies, which requires computational approaches to first align the stacks and then to impute the missing information between slices.

The ability to accurately map and align a 2D section into the 3D tissue context has historically been greatly facilitated by a common coordinate framework as in the case of the Allen Mouse Brain Atlas, but this is largely absent for most human organs. Recently, we have established a spatial mathematical approach, OrganAxis, which defines the position of a spot in space relative to its proximity to defined tissue landmarks. We used this to calculate a continuous cortico-medullary axis of the thymus and integrated spatial data from multiple donors, across spatial technologies and developmental stages [5]. This model and similar ones would be further informed by training on 3D data to minimize loss of information when applied to 2D sections alone.

For the imputation task, some methods perform this as a one-way task (e.g., XFuse [6] or iStar [7]) while others infer a joint latent space for histology and spatial transcriptomics (e.g., BLEEP [8]), but even more powerful analysis models are now being developed, such as full 3D vision-genomics foundation models pre-trained (e.g., via masking) across modalities. Such pre-trained foundation models can be fine-tuned to several downstream tasks (thereby eliminating the need for specialized models for each task in a traditional linear pipeline) and outperform models that did not undergo this pre-training step [9]. By harnessing these data and machine learning tools, molecular connectomics holds the promise of deciphering the molecular basis for emergent properties of cell communities and organs.

Although every part of the human body functions according to a small set of physical and chemical laws, the sheer scale and complexity of molecular interactions in the body leads to phenomena that are not a simple extrapolation of those laws, but are emergent. An example is spontaneous symmetry breaking: in early development the human embryo is bilaterally symmetrical, but this symmetry is subsequently broken, for example, to accommodate the heart and stomach on the left side of the body, and the liver on the right side. However, in about 0.01% of the cases, symmetry breaks in the opposite way, leading to situs inversus, a congenital condition in which all organ positions are left–right transposed, with no obvious medical symptoms. The underlying molecular properties that determine directionality must be defined at the cellular level but remain poorly understood.

At a slightly larger scale, by profiling single cells in the developing thymus and mapping these cells to a continuous organ axis [5], we have observed cytokine gradients that guide T cell development and govern cell motility. Together this leads to the migration of the maturing T cell across the organ as an emergent property that could not have been observed without the broader spatial context.

This example of interactions occurs within the millimeter or centimeter scale. However, many biological phenomena in the human body occur over much larger scales. For example, epithelial cells sense invaders or pathogens via innate danger signals and recruit professional immune cells to mount a systemic immune response, which, after successful clearance of the pathogen, results in whole-body immunity against the pathogen. This whole-body effect is ultimately mediated by molecular cell–cell communication, aided by cell movement across the body, from individual cell activation to the emergent property of immunity.

Linking biology across scales will benefit from generative artificial intelligence and foundation models, and in particular from the unexpected abilities that such models trained on large datasets can acquire [10]. These emergent abilities of foundation models in biology have been largely limited to same-task out-of-distribution tests, but moving towards zero-shot tests across scales, where molecular connectomics could be used for training and benchmarking, would be interesting. For instance, training a foundation model on small-scale (~10 μm) biological data might still enable it to predict biological phenomena that emerge at a larger scale (~1 cm) as an emergent ability.

Studying the molecular connectomics of cell communities will be key to obtaining new insights into organ function. As molecular profiling of single cells expands and is linked to the 3D morphological structures in tissues, emergent properties will become apparent, taking a step closer to connecting the genetic blueprint of the human genome to understanding human physiology, with the cell at the centre of these efforts.
